# Activation of Akt at T308 and S473 in alcohol, tobacco and HPV-induced HNSCC: is there evidence to support a prognostic or diagnostic role?

**DOI:** 10.1186/2162-3619-3-25

**Published:** 2014-10-17

**Authors:** Mohammad R Islam, Ian R Ellis, Michaelina Macluskey, Lynda Cochrane, Sarah J Jones

**Affiliations:** Division of Oral and Maxillofacial Clinical Sciences, The Dental School, University of Dundee, Dundee, DD1 4HR UK; Division of Population Health Science, Medical Research Institute, University of Dundee, Dundee, DD2 4BF UK

**Keywords:** pAkt T308, pAkt S473, HNSCC, Risk factors, Prognosis, Biomarker

## Abstract

**Background:**

Tobacco, alcohol and HPV infection are associated with increased risk of HNSCC. However, little is known about the underlying signaling events influencing risk. We aimed to investigate the relationship between these risk factors and Akt phosphorylation, to determine prognostic value.

**Method:**

VEGF-positive HNSCC biopsies, with known HPV status, were analyzed by immunohistochemistry (IHC) for Akt, phosphorylated at residues S473 and T308. Comparisons between the tissues were carried out using a Mann–Whitney *U* test. Associations between the variables and continuous immunohistochemical parameters were evaluated with general linear models. Patient characteristics and pAkt IHC score were analyzed for possible association with overall survival by Cox proportional hazard models.

**Results:**

Immunohistochemistry revealed that cancer patients had significantly higher levels of pAkt T308 than S473 (P < 0.001). Smoking and alcohol were found to be independent risk factors for Akt phosphorylation at T308 (P = 0.022 and 0.027, respectively). Patients with tumors positive for HPV or pAkt S473 had a poorer prognosis (P = 0.005, and 0.004, respectively). Patients who were heavy drinkers were 49 times more likely to die than non-drinkers (P = 0.003). Patients with low pAkt T308 were more likely to be HPV positive (P = 0.028). Non-drinkers were also found to have lower levels of pAkt T308 and were more likely to have tumors positive for HPV than heavy drinkers (P = 0.044 and 0.007, respectively).

**Conclusion:**

This study suggests different mechanisms of carcinogenesis are initiated by smoking, alcohol and HPV. Our data propose higher phosphorylation of Akt at T308 as a reliable biomarker for smoking and alcohol induced HNSCC progression and higher phosphorylation of Akt at S473 as a prognostic factor for HNSCC.

## Background

HNSCC (Head and Neck Squamous Cell Carcinoma) includes cancers that involve the oral cavity, pharynx and larynx. Each year there are approximately 400 000 cases of cancer of the oral cavity and pharynx, with 160 000 cancers of the larynx, resulting in approximately 300 000 deaths [1]. It is the sixth most common type of cancer worldwide with a five year survival rate of 40-50%, which has shown only moderate improvement over the last two decades [2]. Two modifiable risk factors, tobacco use and alcohol consumption, are thought to explain approximately 75% of the incidence of HNSCC (up to 100 times higher for both) [3]; another important risk factor is HPV infection, which has been detected in around 20% of all cases [4].

Recent studies have focused on the genetic and epigenetic alterations of HNSCC, providing a better understanding of the molecular events underlying the pathogenesis of HNSCC [5, 6]. One of the most frequently altered signaling pathways in HNSCC is the PI3K/Akt cascade [6]. Akt, also known as PKB, is a serine-threonine protein kinase and is central to the phosphatidylinositol 3-kinase (PI3K) signaling pathway [7–9]. PI3K is activated by tyrosine-kinase transmembrane receptors and other signaling intermediates, such as Ras oncogenes and G proteins [10]. PI3K then phosphorylates PtdIns (4,5) P2 (PIP2) yielding PtdIns (3,4,5) P3 (PIP3), which serves as an anchor for intracellular proteins (primarily mediated by pleckstrin homology domains), including Akt amongst others. Membrane-bound Akt is phosphorylated at T308 in the catalytic domain by the kinase PDK1 and at S473 in the regulatory domain by mTORC2 [11, 12]. Full Akt kinase activity is dependent upon phosphorylation at both T308 and S473 residues and this is greatly increased by growth factor receptor signaling [13]. PIP3 is converted back into PIP2 through the action of the lipid phosphatase PTEN, thus terminating the PI3K-initiated signal and avoiding further Akt activation [14]. Growth factor receptor over-expression [15, 16], mutation and down-regulation of PTEN protein [17] and amplification of the PIK3CA gene (the gene coding for the catalytic unit of PI3K) [18] can lead to increased Akt activity. Enhanced Akt activity has indeed been found in 20 to 60% of tumor samples and in the majority of HNSCC-derived cell lines [18–21]. Once Akt is phosphorylated and activated, it is capable of phosphorylating multiple substrates generating diverse cellular processes, such as metabolism, proliferation, survival and protein synthesis [22].

An increasing number of mucosal changes and cellular atypia occur over large areas of the carcinogen-exposed upper aero-digestive tract epithelium, which initiate the stepwise carcinogenesis process in HNSCC. Acquisition of a transformed phenotype and accumulation of specific molecular genetic events are associated with this process [23–28]; yet histopathological evaluation remains the time honored method in risk assessment of carcinoma lesions. In a search for better biological models of risk, Akt activation was recently identified as an early cellular response to carcinogen exposure and may be a significant step in environmental carcinogenesis [29]. Akt activation has also been found to correlate with squamous cell carcinoma progression from normal epithelium to invasive cancer [30].

These observations encouraged us to investigate the role of demographic, pathological and major risk factors (smoking, alcohol and HPV) of HNSCC patients on the activation of Akt (phosphorylation of Akt at Threonine 308 and Serine 473) and to determine their prognostic role.

## Results

### Analysis of patient details

58 HNSCC patient details were analyzed in this study. 33 (57%) were male and 25 (43%) female with ages ranging from 36 to 97 years (median age 64 years). 42 (72%) were smokers and 16 (28%) non-smokers. 7 patients (12%) were non-drinkers, 21 (36%) medium or moderate drinkers and 30 (52%) were heavy drinkers. 31 (53%) patients had negative and 27 (47%) had positive nodal metastasis. Half of the cohort was HPV positive. 43 (74%) patients had T1/T2 and 15 (26%) had T3/T4 tumor size with 5 (9%) of them grade I, 39 (67%) of them grade II and 14 (24%) of them grade III. These data are summarized in Table 1.

**Table 1:** **Demographic, behavioral and pathological data by pAkt status**

		Akt T308 phosphorylation status				Akt S473 phosphorylation status			
	n	High	Medium	Low	p	Medium	Low	None	p
		(n = 40)	(n = 8)	(n = 10)		(n = 5)	(n = 30)	(n = 23)	
		%	%	%		%	%	%	
**Gender**					0.109				0.737
Male	33	62	63	30		60	50	65	
Female	25	38	37	70		40	50	35	
**Age**					0.233				0.376
<65 years	30	50	50	60		80	43	57	
≥65 years	28	50	50	40		20	57	43	
**Location**					0.217				0.094
FOM	10	15	25	20		20	17	17	
RMT	8	10	13	30		0	10	22	
SP	5	10	0	10		40	10	0	
Tong	27	53	50	20		20	47	53	
Alv	4	5	0	20		0	10	4	
Other	4	7	12	0		20	6	4	
**Tumor size**					0.173				0.213
T1-T2	43	70	25	90		60	73	78	
T3-T4	15	30	75	10		40	27	22	
**Grade**					0.523				0.788
I	5	8	12	10		0	7	13	
II	39	65	75	70		80	66	65	
III	14	27	13	20		20	27	22	
**Lymph node metastasis**					0.018				0.327
Positive	27	58	12	30		40	50	43	
Negative	31	42	88	70		60	50	57	
**HPV status**					0.028				0.301
Positive	29	40	63	80		40	43	61	
Negative	29	60	37	20		60	57	39	
**Smoking**					0.022				0.449
Yes	42	82	50	50		80	70	74	
No	16	18	50	50		20	30	26	
**Alcohol**					0.027				0.968
Non-drinker	7	10	0	30		0	17	9	
Medium drinker	21	27	62	40		40	33	39	
Heavy drinker	30	63	38	30		60	50	52	

Note: The General linear model was used for hypothesis testing of the relationship between different variables and pAkt. The P value was obtained from univariable analysis. Percentages represent the column percentages within variable so that the balance between the pAkt groups could be assessed. Abbreviations: *FOM* Floor of the mouth, *RMT* Retromolar trigone, *SP* Soft palate, *Tong* Tongue, *Alv* Alveolus.

### Immunohistochemistry for Akt phosphorylation

**Figure 1:**
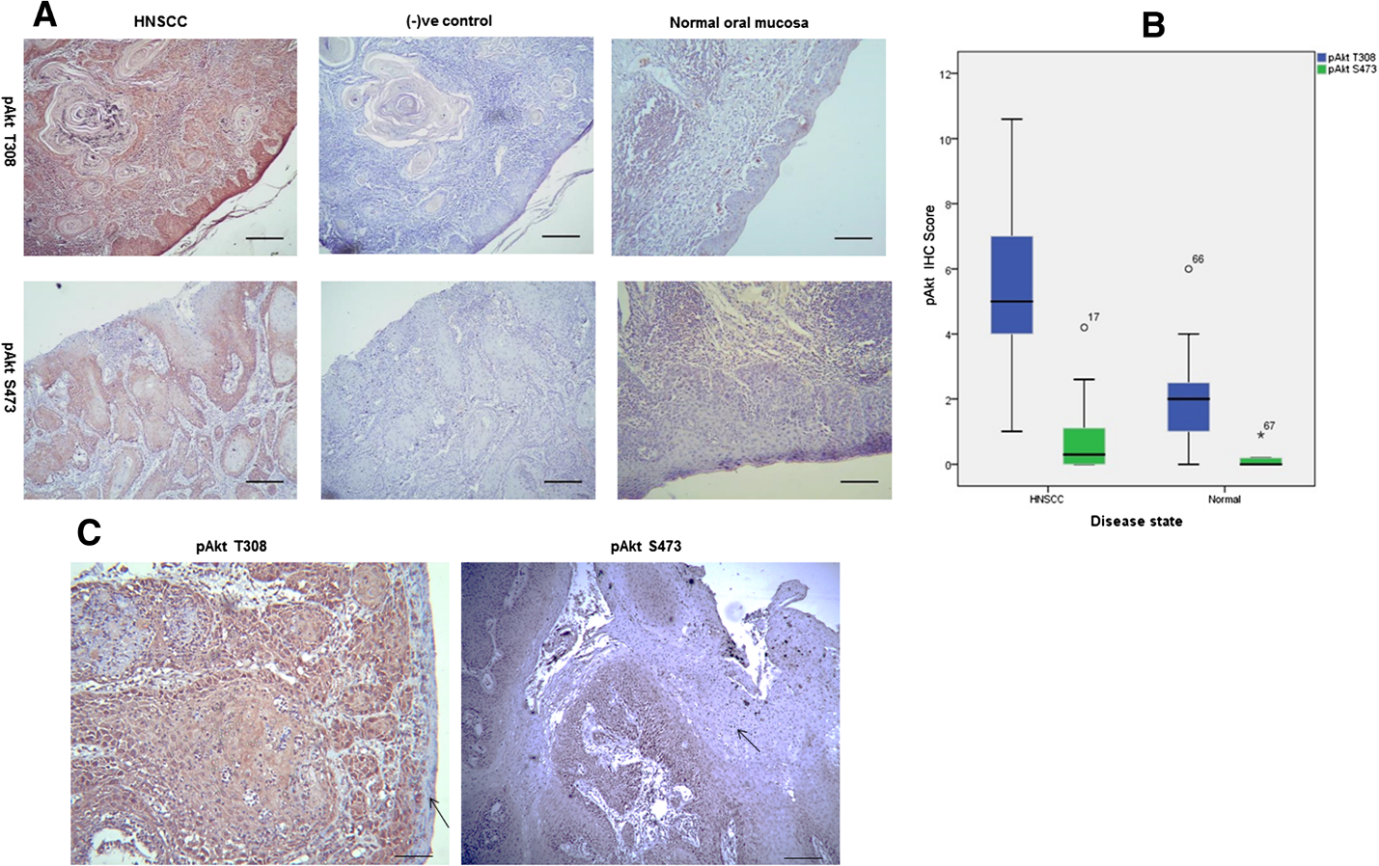
**VEGF positive carcinoma tissues were stained with pAkt antibodies.** Blocking peptides were used to test the efficacy of the antibodies and were tested on duplicate sections of those that had been highly stained for pAkt S473 and pAkt T308 using the antibodies alone. These were used as negative controls. Normal tissues were also used as negative controls, where no staining was observed. All the representative images were taken at x200 magnification except for those stained for pAkt T308, which were taken at x100. **(A)** pAkt S473 and pAkt T308 showed more intense staining in HNSCC tissues compared to normal tissues (P < 0.05). **(B)** Phosphorylation of Akt at residue T308 was found to be significantly higher in HNSCC patient samples compared to phosphorylation at S473 (P < 0.001). **(C)** Phosphorylation status of Akt in non-tumor part of cancer tissues. Very weak or no phosphorylation of Akt at both T308 and S473 was observed in the non-tumor part of HNSCC tissues. The arrow indicates the non-tumor area of the tissues. Images were captured at x200 magnification.

Both normal and VEGF-positive carcinoma patient samples were stained with pAkt S473 and pAkt T308 antibodies. Some samples which were highly stained for pAkt S473 and pAkt T308 were then selected and tested with the blocking peptide for the respective antibody and were used as negative controls (Figure 1A). No staining was observed in the blocking peptide treated tissues and this confirmed the specific binding of the antibodies.

Normal tissue samples were also regarded as negative controls and the level of Akt phosphorylation at T308 was higher in the HNSCC group than the control (median 5.8 vs 2.0, P < 0.001) (Figure 1B). Normal tissue adjacent to the tumors was also tested and the resultant data indicated that there was very low or no pAkt T308/pAkt S473 present (Figure 1C). There is also some evidence to suggest higher levels of pAkt S473 in the cancer group than the controls (P = 0.054) (Figure 1B). There is a statistically significant difference between pAkt T308 and pAkt S473 levels in the cancer patients (pAkt T308, median 5.8 vs pAkt S473, median 0.3, P < 0.001) (Figure 1B). VEGFA is not correlated with pAkt T308 (r = 0.062, P = 0.644) and pAkt S473 (r = 0.181, P = 0.175). Table 1 compares the characteristics between pAkt expression groups. All the samples were found to be phosphorylated at Akt T308 so there is no ‘no phosphorylation’ group for this residue. On the other hand, there is no ‘high phosphorylation’ group for pAkt S473.

### Association of patient characteristics with Akt phosphorylation

Smoking and alcohol were found to be independent risk factors for phosphorylation of Akt at T308 (P = 0.022 and 0.027, respectively) but not for pAkt S473 (P = 0.449 and 0.968, respectively). HNSCC with nodal metastasis was associated with a higher level of pAkt T308 than HNSCC without nodal metastasis (P = 0.018). Smokers and HNSCC with nodal metastasis were found to have higher levels of Akt phosphorylated at T308 than non-smokers (P = 0.022) and HNSCC without modal metastasis (P = 0.018) patients, respectively. HPV-negative patients exhibited higher levels of pAkt T308 compared to those who were HPV positive (P = 0.028). There is some evidence to suggest that heavy drinking patients had higher levels of pAkt T308 than medium drinkers (P = 0.063).

Adjusted by four independent variables, smoking, drinking, nodal status and age, the general linear model accounts for 65.2% (R^2^ = 0.652) of the total variation in pAkt T308 levels (Table 2). This model’s significance statistics for the F-statistic (P < 0.001) indicate that there is only a very small chance that the observed correlation between one or more of the independent variables and the dependent variable is due solely to random sampling error.

**Table 2:** **General Linear Model (Multivariate analysis)**

Independent variables	F	P
Corrected model	4.06	<0.001
Smoking	5.30	0.027
Alcohol	10.56	<0.001
Nodal status	1.31	0.260
Age	4.98	0.031
Smoking * Alcohol	3.20	0.052
Smoking * Nodal status	4.12	0.049
Smoking * Age	0.01	0.919
Alcohol * Nodal status	3.56	0.038
Alcohol * Age	5.31	0.009
Nodal status * Age	2.80	0.102
Smoking * Alcohol * Nodal status	0.17	0.682
Smoking * Alcohol * Age	0.08	0.774
Smoking * Nodal status * Age	6.00	0.019
Alcohol * Nodal status * Age	5.73	0.022
R^2^ = 0.652		

Note: After adjusting Smoking, Alcohol, Nodal Status and Age, this model accounts for 65.2% of the total variations in pAkt T308 level. R^2^ = Coefficient of determination, F = F-statistics.

Dependent variable: pAkt T308 score.

Smoking (P = 0.027), drinking habit (P < 0.001) and age (P = 0.031) showed a significant effect on the phosphorylation of Akt at T308. Alcohol and age, alcohol and nodal involvement, smoking and nodal involvement and smoking and alcohol are strongly correlated with the phosphorylation of Akt at T308 in pairwise combination (P = 0.009, 0.038, 0.049 and 0.052, respectively). Moreover, smoking, nodal involvement, age and alcohol, nodal involvement, age also have a strong correlation with the phosphorylation of Akt T308 (P = 0.019 and 0.022, respectively). None of these independent variables correlated with the phosphorylation of Akt at S473 (univariate and multivariate, P > 0.05).

Non-drinking patients had lower levels of Akt phosphorylated at T308 and were more likely to be infected by HPV than heavy drinkers (*χ*^2^ P = 0.044 and 0.007, respectively). Also, HPV infected patients were shown to have lower levels of Akt phosphorylated at T308 than the non-infected patients (*χ*^2^ P = 0.028).

### Survival Analysis

Levels of pAkt S473 and pAkt T308, as determined by IHC, were examined for association with overall survival using Cox’s proportional hazard model (Table 3). In multivariate analysis, pAkt S473 level, tumor size, alcohol consumption, age and patients’ HPV status had significant effects on overall survival (P = 0.005, 0.005, 0.021, 0.007 and 0.004, respectively).

**Table 3:** **COX proportional hazard model- time to death**

		Unadjusted HR			Adjusted HR		
		HR	95% CI	P	HR	95% CI	P
pAkt S473	Overall			0.168			0.005
	Med:No	6.27	0.87, 45.3	0.069	438	10.9, 1755	0.001
	Med:Low	1.83	0.38, 8.88	0.456	21.5	1.23, 376	0.036
Tumor size	T3/T4:T1/T2	4.59	1.39, 15.2	0.013	15.2	2.28, 102	0.005
Alcohol	Overall			0.005			0.021
	Heavy:Non-drinker	15.4	2.74, 86.7	0.002	49.4	3.04, 801	0.006
	Heavy:Moderate	3.19	0.62, 16.5	0.166	2.69	0.35, 20.8	0.343
Age	≥65:<65	5.41	1.17, 25.05	0.031	46.8	2.81, 781	0.007
HPV	+ve:-ve				89.4	4.05, 1973	0.004
Gender	F:M	1.40	0.42, 4.63	0.581			
Tumor size	Overall			0.310			
Nodal status	+ve: -ve	1.49	0.43, 5.10	0.529			
Smoking	No:Yes	1.45	0.42, 4.95	0.556			
pAkt T308	Overall			0.984			
	Low:High	1.02	0.21, 4.92	0.985			
	Med:High	1.16	0.24, 5.63	0.858			

Note: Unadjusted HR obtained from univariable analysis and adjusted HR from multivariable analysis after adjusting tumor size, alcohol, age, HPV and pAkt S473. All the variables are categorical and HR = exp (B). Abbreviations: *HR* Hazard ratio, 95% *CI* 95% confidence interval.

The rate of deaths in patients with medium levels of phosphorylated Akt S473 was 438 times higher than in those with none and 21.5 times higher than in those with low levels (P = 0.001 and P = 0.036, respectively, Table 3). Patients with tumors of size T3/T4 died more rapidly than patients with tumors T1/T2 (HR = 15.2, P = 0.005). Death rates in heavy drinkers were 49.4 times higher than those who consumed no alcohol (P = 0.006). Older patients (65 years of age or over) were 46.8 times more likely to die than those of 65 years of age (P = 0.007). Finally, HPV positive patients were 89.4 times more likely to die than those with a negative status (P = 0.004).

## Discussion

To our knowledge this is the first report to show the relationships between the major risk factors for HNSCC (alcohol, smoking and HPV) and Akt activation (both at residues T308 and S473) at the protein level using an immunohistochemical staining method (IHC), in surgically resected specimens. Most studies to date have used IHC to assess the prognostic value of Akt activation in HNSCC, but have focused only on phosphorylation of residue S473. As the differential phosphorylation of Akt at the two sites may modulate downstream substrate selectivity and subsequent bioactivity [13], it is not surprising that Akt phosphorylated at any single site could perform certain cellular bioactivities. Two different mechanisms are involved in phosphorylating Akt, therefore overexpression or amplification of any components in these mechanisms may result in over-phosphorylation at any one of the two sites. It is therefore worth studying the phosphorylation status of Akt at both sites in HNSCC specimens to elucidate their different roles.

As only VEGFA positive HNSCC biopsy samples were selected in this study, no statistically significant correlation was found between VEGF and pAkt. The present study showed that Akt was significantly phosphorylated at T308 in VEGF positive HNSCC rather than S473. Alcohol and smoking were positively correlated with pAkt T308 activation but not with pAkt S473. Moreover, Akt activated at T308 showed a significant relationship with lymph node metastasis, which suggests that pAkt T308 may be concerned with invasion and metastasis. This data is similar to our *in vitro* data concerning the migration of tumor cells in response to VEGF, which suggests that migration of oral adeno-squamous cancer cells is dependent on Akt T308 phosphorylation. Our study also disclosed that Akt phosphorylated at both residues controls oral cancer cell motility [31], but it should be remembered that studies performed with cultured cells or tissue models may produce different results. *In vivo*, tumor progression requires both positive and reciprocal feedback between the components of the tissue microenvironment and cancer cells [32].

The activation of Akt in response to alcohol exposure is an important contributor to the molecular effects of excessive alcohol consumption [33]. In 2003, West showed that redundant Akt activation by nicotine and nicotine-derived nitrosamine ketone (NNK) could contribute to tobacco-related carcinogenesis [29]. A study by the Gonzalez group in 2005, revealed that Akt activation was correlated with concomitant PI3K accumulation and PTEN down-regulation in HNSCC, reflecting an early biochemical effect in response to nicotine [18]. Combined with these data, our study supports the basic hypothesis that Akt activation (especially at T308) is a key step in the progression of HNSCC caused by alcohol and smoking. HPV infection, another risk factor for HNSCC, was found to be negatively correlated with Akt activation at T308, as HPV positive HNSCC patients showed lower levels of pAkt T308. Non-drinking patients had lower levels of activated Akt at T308 too and there were more HPV positive patients among non-drinkers than amongst the heavy drinkers. Earlier epidemiologic research supports this data, that is non-smokers and light or non-drinkers are more likely to have tumors positive for HPV than are heavy smokers and drinkers [34]. Molinolo *et al.* [35] showed in their study that HPV positive HNSCC patients over-activate Akt at S473 and mTOR. Although we have not found any association between HPV infection and pAkt S473 activation, this may suggest that there are two different mechanisms of cancer progression initiated by alcohol, smoking and HPV. The Kelsey group study (2007), strongly supports the emerging view that the etiology of HPV related HNSCC is distinct from that of HNSCC tumors associated with smoking and drinking [36]. Increased Akt activation at T308 by excessive alcohol and smoking may be responsible for cancer development and progression, including metastasis, whereas HNSCC by HPV infection may over-activate Akt at S473 and be responsible for poor survival.

In this study we show that increased pAkt S473 levels in HNSCC are a strong predictor for poor patient outcome. In the multivariate Cox proportional hazard model, adjusted for well recognized prognostic indicators (e.g. tumor size and age), pAkt S473 status remained a strong predictor. This is corroborated by three other studies which have shown that pAkt activated at S473 is associated with poor prognosis in oral cancer [37–39]. Although further molecular analysis is needed to investigate the mechanism of smoking and alcohol related HNSCC development, we can propose pAkt T308 as a reliable biomarker for smoking and alcohol induced HNSCC progression.

## Conclusions

The predictive role of Akt activation in HNSCC suggests that targeting PI3K/Akt and mTORC2/Akt pathway along with RTK (receptor tyrosine kinase) might be a useful strategy for therapy in this disease. A large cohort with a longer follow-up of pre-neoplastic and HNSCC lesions is needed to more accurately define the role of Akt activation in carcinogenesis and to integrate this data into a risk model for carcinoma development and progression. In conclusion, our findings suggest that targeting Akt activation might be of interest as part of a combination therapy in HNSCC, as described earlier [40].

## Methods

### Patients

Ethical approval (ID: LEC271/03) was granted for the prospective collection of tissues which were stored at the Tayside Tissue Bank. In total 64 HNSCC and 11 normal oral mucosal tissues (from non-tumor patients) were collected from patients treated at Ninewells Hospital Tayside. Baseline data obtained from patient charts included age, sex, histology, site, drinking and smoking status, nodal involvement, survival and follow-up data. Patients were followed-up for a total of 66 months (median, 40 months) after diagnosis.

Some continuous variables (such as age, drinking and smoking status) were changed into categorical variables in this study with clear justification (statistical and/or clinical reasons). Age was grouped as ‘<65 years of age’ and ‘≥65 years’ because approximately 50% patients were above/below 65 and a growing number of patients with Head and Neck Squamous Cell Carcinoma (HNSCC) are aged 65 and older [41]. Patients who consumed alcohol were referred to as ‘drinkers’ throughout this article. Drinkers were categorized as non-drinkers, medium or moderate drinkers (less than 7 units per week for women and 14 units per week for men or occasional or social, regarded as low risk group by NIAAA), and heavy drinkers (over 7 units per week for women and 14 units per week for men) according to the National Institute of Alcohol Abuse and Alcoholism [42]. Smoking status was classified as non- or light smokers (less than 5 cigarettes per day) and smokers (more than 5 cigarettes per day) after reviewing the literature [43–45].

HNSCC tissues were stained for VEGFA expression by IHC [31] and tissues with IHC scores of more than 3 were selected and regarded as positive. Tissues were also analyzed for HPV DNA by PCR, automated DNA sequencing and the SPF_10_-LiPA_25_ method [46].

### Immunohistochemistry

The paraffin-embedded tissues were cut into 5 μm sections, dewaxed in xylene and then rehydrated in serial ethanol solutions, before washing in distilled water for 5 minutes. 58 VEGFA positive HNSCC and 11 normal mucosal samples were then probed with pAkt T308 (#2965) and pAkt S473 (#4060) antibodies according to the manufacturer’s instructions (Cell Signaling Technology Inc., Danvers, MA, USA). In brief, after the deparaffinization and rehydration process, antigens were unmasked by boiling in 10mM Sodium citrate buffer (pH 6.0) using a microwave, followed by maintenance at a sub-boiling temperature for 10 minutes and then cooling for 30 minutes on the bench top. 3% (v/v) H_2_O_2_ was then used as a peroxidase blocker and TBST (Tris buffered saline with 0.1% v/v Tween 20) for washing. Sections were then blocked with 5% (v/v) normal goat serum (NGS) plus TBST for 1 hour at room temperature. Sections were then incubated with antibodies against pAkt S473 (1:50) and pAkt T308 (1:50) diluted in 5% (v/v) NGS/TBST in a humidified chamber overnight at 4°C. After equilibration, sections were then washed three times with TBST and then incubated in signal stain boost detection reagent (HRP, rabbit #8114, Cell Signaling Technology) for 30 minutes at room temperature. Visualization was achieved by incubation with 3,3’-diaminobenzidine (DAB) (Sigma-Aldrich, MO, USA) for 5 minutes and counterstaining with Mayer’s haematoxylin (Sigma) and eosin. Rehydration and mounting processes were then followed as described in the instruction manual (Cell Signaling Technology). Normal oral mucosal tissues were used as negative controls. The pAkt S473 and pAkt T308 antibodies were blocked using the respective blocking peptide (#1140 and 1145B, respectively, Cell Signaling Technology) by adding twice the volume of peptide as volume of antibody used, in a total volume of 100 μl. These tissues were also used as negative control.

### IHC score

According to the scoring systems that have been reported previously in the literature, [47, 48] with some modifications, pAkt staining scoring was performed as follows: stained sections were visualized using a light microscope at high power field and were evaluated by three observers without prior knowledge of the patients’ characteristics. An intra-class correlation (inter-observer correlation) analysis using a mixed model and testing for consistency gave a Chronbach’s alpha of more than 0.8. The cells showing cytoplasmic and/or nuclear staining were judged as positive. Five high power fields were selected randomly under the microscope. The average percentage of positive staining was calculated for each field. The average percentage of tissue staining was designated as 0 when less than 10% was stained, 1 when 10-25%, 2 when 25-50%, 3 when 50-75% and 4 when >75% of tissues stained. The intensity of tissue staining positively was categorized as follows: 0, no appreciable staining in tissues; 1, barely detectable staining as compared with stromal elements; 2, readily appreciable brown staining distinctly marking cell cytoplasm and/or nucleus; and 3, dark brown staining in tissues completely obscuring cytoplasm and/or nucleus. Scoring was performed according to the product of staining intensity and average percentage of tissue staining positively ranging from 0–12. In the following analysis, the level of Akt phosphorylation was evaluated using the pAkt index either as a continuous variable directly or categorized as no phosphorylation (IHC score 0), low phosphorylation (IHC score 0.1-2.0), medium phosphorylation (IHC score 2.1-5.0), high phosphorylation (IHC score 5.1-12.0) after reviewing a number of studies [37, 49–60].

### Statistics

Data were analyzed using the statistical package IBM SPSS 19.0. Comparisons between the tissues (HNSCC and normal) regarding the Akt phosphorylation were carried out using a Mann–Whitney *U* test. Associations between categorical demographic, pathological and behavioral factors were investigated using cross tabulation and Pearson chi-square test. Associations between these variables and continuous immunohistochemical parameters were evaluated with general linear models (both univariate and multivariate). Bonferroni’s correction for multiple comparisons was applied where appropriate.

Patients’ characteristics and pAkt IHC score were analyzed for possible association with overall survival by univariate and multivariate Cox proportional hazard models. Overall survival was defined as the time between diagnosis date and death or last follow-up date. Initially, explanatory factors were screened for univariate associations with death, using a method appropriate to the distribution of the data. If the two-sided P value was <0.300 for any variable it was considered as a candidate in multiple regression models (Hosmer-Lemeshow criterion). Variables with P ≥ 0.300 were discarded at this stage. The assumption of proportional hazards was checked for independent variables by plotting the logarithm of the cumulative hazards functions. Starting with the set of variables identified for inclusion from the previous steps, a multiple Cox regression model was built using a step-wise approach.

All tests were two-sided, using the 5% significance level.

## Abbreviations

HNSCC: Head and neck squamous cell carcinoma
pAkt T308: Akt phosphorylated at Threonine 308
pAkt S473: Akt phosphorylated at Serine 473
VEGF: Vascular endothelial growth factor
HPV: Human papilloma virus.

## References

[1] Boyle P, Levin B (2008). World Cancer Report 2008.

[2] Al-Sarraf M (2002). Treatment of locally advanced head and neck cancer: historical and critical review. Cancer Control.

[3] Neville BW, Day TA (2002). Oral cancer and precancerous lesions. CA Cancer J Clin.

[4] Gillison ML, Castellsague X, Chaturvedi A, Goodman MT, Snijders P, Tommasino M, Arbyn M, Franceschi S (2013). Comparative epidemiology of HPV infection and associated cancers of the head and neck and cervix. Int J Cancer.

[5] Mao L, Hong WK, Papadimitrakopoulou VA (2004). Focus on head and neck cancer. Cancer Cell.

[6] Tan M, Myers JN, Agrawal N (2013). Oral cavity and oropharyngeal squamous cell carcinoma genomics. Otolaryngol Clin North Am.

[7] Bellacosa A, Kumar CC, Di Cristofano A, Testa JR (2005). Activation of AKT kinases in cancer: implications for therapeutic targeting. Adv Cancer Res.

[8] Altomare DA, Guo K, Cheng JQ, Sonoda G, Walsh K, Testa JR (1995). Cloning, chromosomal localization and expression analysis of the mouse Akt2 oncogene. Oncogene.

[9] Fresno Vara JA, Casado E, de Castro J, Cejas P, Belda-Iniesta C, Gonzalez-Baron M (2004). PI3K/Akt signalling pathway and cancer. Cancer Treat Rev.

[10] Rodriguez-Viciana P, Warne PH, Dhand R, Vanhaesebroeck B, Gout I, Fry MJ, Waterfield MD, Downward J (1994). Phosphatidylinositol-3-OH kinase as a direct target of Ras. Nature.

[11] Alessi DR, Cohen P (1998). Mechanism of activation and function of protein kinase B. Curr Opin Genet Dev.

[12] Sarbassov DD, Guertin DA, Ali SM, Sabatini DM (2005). Phosphorylation and regulation of Akt/PKB by the rictor-mTOR complex. Science.

[13] Bozulic L, Hemmings BA (2009). PIKKing on PKB: regulation of PKB activity by phosphorylation. Curr Opin Cell Biol.

[14] Song MS, Salmena L, Pandolfi PP (2012). The functions and regulation of the PTEN tumour suppressor. Nat Rev Mol Cell Biol.

[15] Sweeny L, Zimmermann TM, Liu Z, Rosenthal EL (2012). Evaluation of tyrosine receptor kinases in the interactions of head and neck squamous cell carcinoma cells and fibroblasts. Oral Oncol.

[16] Thariat J, Etienne-Grimaldi MC, Grall D, Bensadoun RJ, Cayre A, Penault-Llorca F, Veracini L, Francoual M, Formento JL, Dassonville O, De Raucourt D, Geoffrois L, Giraud P, Racadot S, Moriniere S, Milano G, Van Obberghen-Schilling E (2012). Epidermal growth factor receptor protein detection in head and neck cancer patients: a many-faceted picture. Clin Cancer Res.

[17] Squarize CH, Castilho RM, Abrahao AC, Molinolo A, Lingen MW, Gutkind JS (2013). PTEN deficiency contributes to the development and progression of head and neck cancer. Neoplasia.

[18] Pedrero JM, Carracedo DG, Pinto CM, Zapatero AH, Rodrigo JP, Nieto CS, Gonzalez MV (2005). Frequent genetic and biochemical alterations of the PI 3-K/AKT/PTEN pathway in head and neck squamous cell carcinoma. Int J Cancer.

[19] Amornphimoltham P, Patel V, Molinolo A, Gutkind JS, Glick AB, Van Waes C (2011). Head and Neck Cancer and PI3K/Akt/mTOR Signaling Network: Novel Molecular Targeted Therapy. Signaling Pathways in Squamous Cancer.

[20] Mandal M, Younes M, Swan EA, Jasser SA, Doan D, Yigitbasi O, McMurphey A, Ludwick J, El-Naggar AK, Bucana C, Mills GB, Myers JN (2006). The Akt inhibitor KP372-1 inhibits proliferation and induces apoptosis and anoikis in squamous cell carcinoma of the head and neck. Oral Oncol.

[21] Moral M, Paramio JM (2008). Akt pathway as a target for therapeutic intervention in HNSCC. Histol Histopathol.

[22] Lindsley CW (2010). The Akt/PKB family of protein kinases: a review of small molecule inhibitors and progress towards target validation: a 2009 update. Curr Top Med Chem.

[23] Grandis JR, Drenning SD, Zeng Q, Watkins SC, Melhem MF, Endo S, Johnson DE, Huang L, He Y, Kim JD (2000). Constitutive activation of Stat3 signaling abrogates apoptosis in squamous cell carcinogenesis in vivo. Proc Natl Acad Sci USA.

[24] Liotta LA, Steeg PS, Stetler-Stevenson WG (1991). Cancer metastasis and angiogenesis: an imbalance of positive and negative regulation. Cell.

[25] Mao L, Lee JS, Fan YH, Ro JY, Batsakis JG, Lippman S, Hittelman W, Hong WK (1996). Frequent microsatellite alterations at chromosomes 9p21 and 3p14 in oral premalignant lesions and their value in cancer risk assessment. Nat Med.

[26] Papadimitrakopoulou VA, Izzo J, Mao L, Keck J, Hamilton D, Shin DM, El-Naggar A, den Hollander P, Liu D, Hittelman WN, Hong WK (2001). Cyclin D1 and p16 alterations in advanced premalignant lesions of the upper aerodigestive tract: role in response to chemoprevention and cancer development. Clin Cancer Res.

[27] Rosin MP, Cheng X, Poh C, Lam WL, Huang Y, Lovas J, Berean K, Epstein JB, Priddy R, Le ND, Zhang L (2000). Use of allelic loss to predict malignant risk for low-grade oral epithelial dysplasia. Clin Cancer Res.

[28] Slaughter DP, Southwick HW, Smejkal W (1953). Field cancerization in oral stratified squamous epithelium; clinical implications of multicentric origin. Cancer.

[29] West KA, Brognard J, Clark AS, Linnoila IR, Yang X, Swain SM, Harris C, Belinsky S, Dennis PA (2003). Rapid Akt activation by nicotine and a tobacco carcinogen modulates the phenotype of normal human airway epithelial cells. J Clin Invest.

[30] Amornphimoltham P, Sriuranpong V, Patel V, Benavides F, Conti CJ, Sauk J, Sausville EA, Molinolo AA, Gutkind JS (2004). Persistent activation of the Akt pathway in head and neck squamous cell carcinoma: a potential target for UCN-01. Clin Cancer Res.

[31] Islam MR, Jones SJ, Macluskey M, Ellis IR (2014). Is there a pAkt between VEGF and oral cancer cell migration?. Cell Signal.

[32] Cirri P, Chiarugi P (2011). Cancer associated fibroblasts: the dark side of the coin. Am J Cancer Res.

[33] Neasta J, Ben Hamida S, Yowell QV, Carnicella S, Ron D (2011). AKT signaling pathway in the nucleus accumbens mediates excessive alcohol drinking behaviors. Biol Psychiatry.

[34] Lindel K, Beer KT, Laissue J, Greiner RH, Aebersold DM (2001). Human papillomavirus positive squamous cell carcinoma of the oropharynx: a radiosensitive subgroup of head and neck carcinoma. Cancer.

[35] Molinolo AA, Marsh C, El Dinali M, Gangane N, Jennison K, Hewitt S, Patel V, Seiwert TY, Gutkind JS (2012). mTOR as a molecular target in HPV-associated oral and cervical squamous carcinomas. Clin Cancer Res.

[36] Applebaum KM, Furniss CS, Zeka A, Posner MR, Smith JF, Bryan J, Eisen EA, Peters ES, McClean MD, Kelsey KT (2007). Lack of association of alcohol and tobacco with HPV16-associated head and neck cancer. J Natl Cancer Inst.

[37] Massarelli E, Liu DD, Lee JJ, El-Naggar AK, Lo Muzio L, Staibano S, De Placido S, Myers JN, Papadimitrakopoulou VA (2005). Akt activation correlates with adverse outcome in tongue cancer. Cancer.

[38] Yu Z, Weinberger PM, Sasaki C, Egleston BL, Speier WF, Haffty B, Kowalski D, Camp R, Rimm D, Vairaktaris E, Burtness B, Psyrri A (2007). Phosphorylation of Akt (Ser473) predicts poor clinical outcome in oropharyngeal squamous cell cancer. Cancer Epidemiol Biomarkers Prev.

[39] Lim J, Kim JH, Paeng JY, Kim MJ, Hong SD, Lee JI, Hong SP (2005). Prognostic value of activated Akt expression in oral squamous cell carcinoma. J Clin Pathol.

[40] LoPiccolo J, Blumenthal GM, Bernstein WB, Dennis PA (2008). Targeting the PI3K/Akt/mTOR pathway: effective combinations and clinical considerations. Drug Resist Updat.

[41] VanderWalde NA, Meyer AM, Liu H, Tyree SD, Zullig LL, Carpenter WR, Shores CD, Weissler MC, Hayes DN, Fleming M, Chera BS (2013). Patterns of care in older patients with squamous cell carcinoma of the head and neck: a surveillance, epidemiology, and end results-medicare analysis. J Geriatr Oncol.

[42] *Moderate and Binge Drinking*. [http://www.niaaa.nih.gov/alcohol-health/overview-alcohol-consumption/moderate-binge-drinking]

[43] Fagan P, Rigotti NA (2009). Light and intermittent smoking: the road less traveled. Nicotine Tob Res.

[44] Husten CG (2009). How should we define light or intermittent smoking? does it matter?. Nicotine Tob Res.

[45] Shiffman S (2009). Light and intermittent smokers: background and perspective. Nicotine Tob Res.

[46] Sailan AT (2010). HPV and p16 in head and neck cancer. PhD Thesis.

[47] Malik SN, Brattain M, Ghosh PM, Troyer DA, Prihoda T, Bedolla R, Kreisberg JI (2002). Immunohistochemical demonstration of phospho-Akt in high Gleason grade prostate cancer. Clin Cancer Res.

[48] Tang J-M, He Q-Y, Guo R-X, Chang X-J (2006). Phosphorylated Akt overexpression and loss of PTEN expression in non-small cell lung cancer confers poor prognosis. Lung Cancer.

[49] Bose S, Chandran S, Mirocha JM, Bose N (2006). The Akt pathway in human breast cancer: a tissue-array-based analysis. Mod Pathol.

[50] Glynn S, Prueitt R, Ridnour L, Boersma B, Dorsey T, Wink D, Goodman J, Yfantis H, Lee D, Ambs S (2010). COX-2 activation is associated with Akt phosphorylation and poor survival in ER-negative, HER2-positive breast cancer. BMC Cancer.

[51] Kirkegaard T, Witton CJ, McGlynn LM, Tovey SM, Dunne B, Lyon A, Bartlett JM (2005). AKT activation predicts outcome in breast cancer patients treated with tamoxifen. J Pathol.

[52] Lim WT, Zhang WH, Miller CR, Watters JW, Gao F, Viswanathan A, Govindan R, McLeod HL (2007). PTEN and phosphorylated AKT expression and prognosis in early- and late-stage non-small cell lung cancer. Oncol Rep.

[53] Messersmith W, Oppenheimer D, Peralba J, Sebastiani V, Amador M, Jimeno A, Embuscado E, Hidalgo M, Iacobuzio-Donahue C (2005). Assessment of Epidermal Growth Factor Receptor (EGFR) signaling in paired colorectal cancer and normal colon tissue samples using computer-aided immunohistochemical analysis. Cancer Biol Ther.

[54] Ogino S, Meyerhardt JA, Cantor M, Brahmandam M, Clark JW, Namgyal C, Kawasaki T, Kinsella K, Michelini AL, Enzinger PC (2005). Molecular alterations in tumors and response to combination chemotherapy with gefitinib for advanced colorectal cancer. Clin Cancer Res.

[55] Scartozzi M, Giampieri R, Maccaroni E, Mandolesi A, Biagetti S, Alfonsi S, Giustini L, Loretelli C, Faloppi L, Bittoni A, Bianconi M, Del Prete M, Bearzi I, Cascinu S (2012). Phosphorylated AKT and MAPK expression in primary tumours and in corresponding metastases and clinical outcome in colorectal cancer patients receiving irinotecan-cetuximab. J Transl Med.

[56] Schmitz KJ, Grabellus F, Callies R, Otterbach F, Wohlschlaeger J, Levkau B, Kimmig R, Schmid KW, Baba HA (2005). High expression of focal adhesion kinase (p125FAK) in node-negative breast cancer is related to overexpression of HER-2/neu and activated Akt kinase but does not predict outcome. Breast Cancer Res.

[57] Stal O, Perez-Tenorio G, Akerberg L, Olsson B, Nordenskjold B, Skoog L, Rutqvist LE (2003). Akt kinases in breast cancer and the results of adjuvant therapy. Breast Cancer Res.

[58] Tokunaga E, Kimura Y, Mashino K, Oki E, Kataoka A, Ohno S, Morita M, Kakeji Y, Baba H, Maehara Y (2006). Activation of PI3K/Akt signaling and hormone resistance in breast cancer. Breast Cancer.

[59] Wu Y, Mohamed H, Chillar R, Ali I, Clayton S, Slamon D, Vadgama J (2008). Clinical significance of Akt and HER2/neu overexpression in African-American and Latina women with breast cancer. Breast Cancer Res.

[60] Zhang W, Hart J, McLeod HL, Wang HL (2005). Differential expression of the AP-1 transcription factor family members in human colorectal epithelial and neuroendocrine neoplasms. Am J Clin Pathol.

